# High Quality Bioreplication of Intricate Nanostructures from a Fragile Gecko Skin Surface with Bactericidal Properties

**DOI:** 10.1038/srep41023

**Published:** 2017-01-25

**Authors:** David William Green, Kenneth Ka-Ho Lee, Jolanta Anna Watson, Hyun-Yi Kim, Kyung-Sik Yoon, Eun-Jung Kim, Jong-Min Lee, Gregory Shaun Watson, Han-Sung Jung

**Affiliations:** 1 Division in Anatomy and Developmental Biology, Department of Oral Biology, Oral Science Research Center, BK21 PLUS Project, Yonsei University College of Dentistry, Seoul, Korea; 2 MOE Key Laboratory for Regenerative Medicine, School of Biomedical Sciences, Faculty of Medicine, The Chinese University of Hong Kong, Shatin, Hong Kong, SAR; 3 University of the Sunshine Coast, School of Science & Engineering, Sippy Downs, QLD 4558, Australia; 4 Oral Biosciences, Faculty of Dentistry, The University of Hong Kong, Sai Ying Pun, Hong Kong, SAR

## Abstract

The external epithelial surfaces of plants and animals are frequently carpeted with small micro- and nanostructures, which broadens their adaptive capabilities in challenging physical habitats. Hairs and other shaped protuberances manage with excessive water, light contaminants, predators or parasites in innovative ways. We are interested in transferring these intricate architectures onto biomedical devices and daily-life surfaces. Such a project requires a very rapid and accurate small-scale fabrication process not involving lithography. In this study, we describe a simple benchtop biotemplating method using shed gecko lizard skin that generates duplicates that closely replicate the small nanotipped hairs (spinules) that cover the original skin. Synthetic replication of the spinule arrays in popular biomaterials closely matched the natural spinules in length. More significantly, the shape, curvature and nanotips of the synthetic arrays are virtually identical to the natural ones. Despite some small differences, the synthetic gecko skin surface resisted wetting and bacterial contamination at the same level as natural shed skin templates. Such synthetic gecko skin surfaces are excellent platforms to test for bacterial control in clinical settings. We envision testing the biocidal properties of the well-matched templates for fungal spores and viral resistance in biomedicine as well as co/multi-cultures.

There are many surface regions of animals and plants with embellishments for complex functions that allow for successful interactions with the physical world^1^. In some cases, these surface regions are hierarchical in structure and are often new to science and technology. Fascinatingly, most evolutionary adaptations have had no analogue in technology (until they are discovered in nature) and they frequently have a superior performance to even the best human innovations. Some of the most significant surface solutions can be transferred from nature to technology, to address important interfacial challenges, such as, anti-wetting (e.g., gecko lizard skins)^2^, self-cleaning^3^, antireflection^4^, omniphobicity^5^,^6^ (e.g., springtail cuticles), icephobicity^7^ and wet and dry reversible adhesion^8^,^9^,^10^,^11^.

Many epithelial surface structures have technological potential across biomedicine, including drug-delivering skin plasters^12^, subcutaneous tissue tapes^13^ and gene transfer devices^14^, as well as an influence over stem cell differentiation, biosensing platforms, lab-on-chip templates and organoid induction. Two of the most sought after physical properties among biological surface systems are decontamination and antibiofouling^15^, which are conducive to human cell attachment^2^. These properties could have a profound impact on medical systems technologies that are used to fight antibiotic resistant bacteria (e.g., implants, catheters, drains, intravenous lines and ports). In the field of biology, applications of micro- and nano-machined surface systems or fluidic channels could lead to exquisite control over eukaryote and prokaryote cell shape function and their growing environment^17^,^18^.

Engineered surfaces bearing tiny structures at the nanoscale are typically made with techniques translated from the semiconductor and electronics industries, such as electron and ion-based lithography^19^, hard lithography^20^, photo and soft lithography^16^, which can duplicate very small features to below 50 nm in dimensions. Biotemplating methodologies remain at the forefront of efforts to duplicate complex biomolecular microscopic and macroscopic structures and architectures; DNA & subcellular microtubules, Tobacco mosaic viruses^21^, Diatoms^22^, beetle carapaces, insect wings and insect compound eyes^23^,^24^. Casting material onto a natural structure generates a negative mold, which is then used as a template for additional casting with a desired material, producing a duplicate of the original^25^. Casting and molding have been successfully used to copy plant leaf surfaces (e.g. lotus leaf) and insect wings^26^.

One taxonomic group of lizards, the gecko, has caught our attention because of the superhydrophobicity and unique design of their hair structures and associated topography, which have profound antimicrobial action^2^. These hair-like protuberances develop across the entire body, and interestingly, when the gecko animal sheds its skin, the structures remain perfectly intact^27^,^28^. They are 1–4 microns in length and taper to a sub 50-nm nanotip, and thus consist of two length scales. Towards the tip, the spinule can curve significantly. The spinules are elastic and flexible, which may be an important and unique element in killing bacteria. The flexible stature, integrated microscopic and nanoscopic elements and curved shape make these structures difficult to copy directly with any reasonable degree of precision and accuracy.

To effectively exploit the performance of gecko spinules in self-cleaning and decontamination for healthcare materials, a method was needed which could facilitate replication of the correct spinule length, curvature and nanotip design. We worked under the assumption, which does not always hold true, that to mimic function it is necessary to copy the structure almost perfectly. In this study, we adapted a simple biotemplating methodology based on the casting and molding strategy to generate excellent copies of spinules onto materials measuring up to 10 × 10 cm (for some species).

While printing and imprinting techniques, plotted by 3D digital imaging possess the capability to replicate surface features with unsurpassed levels of accuracy and precision, in this study we favoured the benchtop procedure to generate structures that can be rapidly employed in laboratory testing and evaluation studies.

## Results

**Figure 1:**
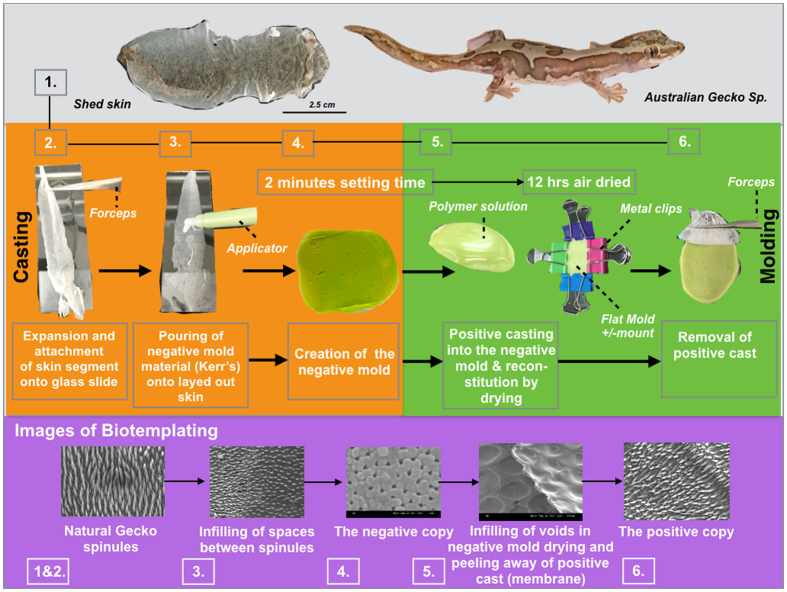
A flow diagram showing the biotemplating methodology for duplicating gecko skin spinules into a small range of biopolymer membranes. Shed gecko skin was immobilized on a film of water that additionally inflated the spinules into their natural state as seen in the living gecko. Whole shed skin from a single gecko lizard (7–10 cm in length) [step 1] was expanded onto a thin film of water covering a glass microscope slide and dried in place [step 2]. The now flat panel of skin was casted with PVS using a plastic applicator [step 3]. The PVS was hardened within 2 minutes and generated a negative mold [step 4]. The gecko skin was peeled away from the PVS membrane. A polymer solution was added to the gecko skin patterned face of the PVS membrane [step 5]. The polymer solution was air dried for 12 hours and, for replicas made with biomaterial cross-linking agents were added *in situ*. All membranes were air dried for 1–2 hours prior to use. The positive casted membranes were peeled away from the PVS mold [step 6]. The corresponding Biotemplating SEM images of the various replication stages are also shown.

Shed gecko skin is a wafer-thin exterior layer of fresh skin (the last layers of the integument) that becomes distorted following molting (see Fig. 1 (step 1)). The shed skin conformation may arise due to the heavily folded regions (troughs) in between scales, which we have previously speculated may be required to accommodate movement of the lizard which can adopt significant body conformations^28^. The topography may also deflate to some extent presumably because of a lack of hydrostatic pressure from the underlying living tissue. However, the physical properties and elastic nature of the chemistry including β-keratin material allows the spinules to maintain their structure. This structural preservation was highlighted, in a study by Vucko *et al*.^29^, in which replicates were generated directly from living geckos and retained their intrinsic structure.

So far, we have focused on analyzing spinule morphology and have not considered other elements that could also contribute to the decontamination functions of the skin. One of these unconsidered elements is the internal material design of individual spinules. From SEM images of truncated spinules, there was no evidence of an internal structure that would indicate a mechanical or sensory function. However, in a recent study investigating the adhesion system of beetle tarsal setae, there was a gradient of different materials built inside these protuberances^30^. We have focused on examination of the spinule replication as opposed to hairs associated with other body regions for our study (e.g. the lenticular epidermal sense organ).

### Preparation of Gecko skins for casting

Compared to the outer exoskeleton surface structure of many insect cuticles (which have also been exploited for their optical and antibacterial properties), gecko skin epidermis is relatively frail for many species. It is also difficult to handle the skin in the laboratory because it is easily torn. Due to this fragility, in combination with the shedding process, the shed skin can often assume a wrinkled, almost origami-like orientation, the molding techniques are liable to damage natural skin. We have exploited the polar nature and surface tension of water to *orientate, unravel* and *fix* the reptilian skin to a glass microscope slide, which allowed us to apply polyvinylsiloxane (PVS) material uniformly with relative ease and cause no damage to the skin. One of the main concerns arising from this casting was the possibility of damaging the spinules. It was also possible that the skin would adhere to the mold, but when the skin was viewed under Scanning Electron Microscopy (SEM), the negative PVS molds were completely free of gecko skin material (Supplementary Fig. S1).

### Fabrication of positive gecko skin molds

**Figure 2:**
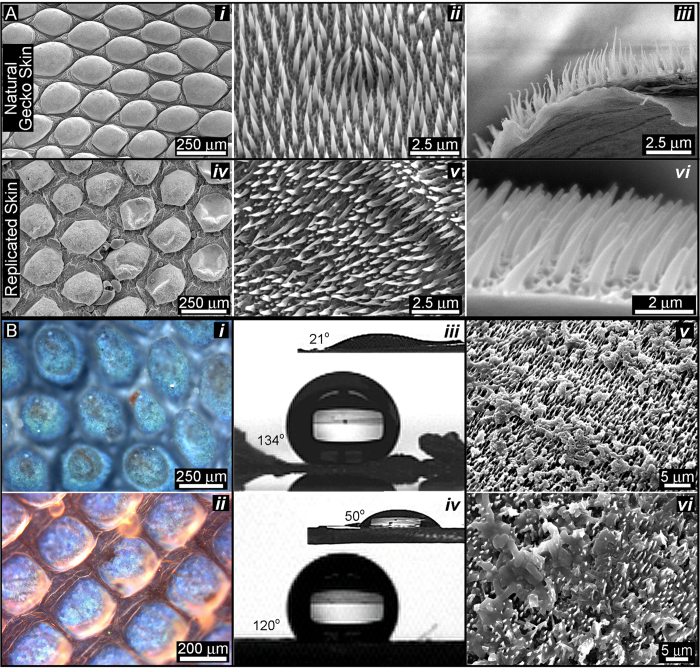
The similarities of spinule structure and selected function between polystyrene replicas and natural gecko. (**A**) SEM surface view of the natural shed gecko skin surface at increasing levels of scale (A*i*–*iii*) versus a polystyrene replica of the same gecko skin (A*iv*–*vi*) (*Strophurus williamsi* gecko), which was also visualized and digitally defined using laser confocal microscopy in Supplementary Fig. S7; (A*iii* and *vi*) SEM cross sectional view of the gecko skin surface versus a polystyrene replica reveals the similarity of the spinule length (3–4 μm); (**B***i*) the natural gecko skin surface viewed under polarized white light to show its characteristic birefringence due to the ordering of the spinules, which caused double refraction; (B*ii*) the polystyrene replica viewed under differential interference contrast microscopy. This image shows the intrinsic birefringence under polarized light observed in the natural skin material, which showed the same arrangement for the spinules. The functional effects of the replica versus the natural gecko skin spinule array are shown in B*iii*–*vi*; (B*iii*) A high-power image of a single, sessile distilled water droplet (25 μL) placed on top of a small segment of natural shed gecko skin (*Strophurus williamsi*) versus a water droplet placed on a replica polystyrene membrane (B*iv*). The insets in B*iii* and *iv* represent the smooth control surfaces for beta keratin and polystyrene, respectively. (B*v*, B*vi*) SEM images of multi-colonies (8 non-competitive strains) of gut bacteria cultivated on the natural gecko skin surface (Hitachi SEM S-3000N); (B*v*) and replica (B*vi*) demonstrate the similar bactericidal function.

Positive molds are fabricated by permeating the negative mold with biopolymer solutions. We have shown that 5%+ solutions of chitosan, alginate or silk fibroin can infiltrate the tiny spaces and harden, spontaneously *in situ* without excessive swelling (Fig. 1 (step 5)), which we determined by the lack of shape distortions in the mold and synthetic spinules. The biopolymers were dried in place within the mold. The generated biopolymer film was then peeled away with forceps to reveal a positive, replicated spinule array (Fig. 1 (step 6)). In our ongoing studies to control eukaryotic cells with gecko nanotopography, we first cast negative gecko molds in tissue culture plastic polystyrene. Our first objective was to fabricate thin membranes that were covered with duplicated gecko spinules (Fig. 2A, B*i, ii*) followed by the tests for self-cleaning and biocidal function (Fig. 2B*iii, iv, v, vi*). We extended the molding to include other healthcare relevant biopolymers that have the potential to service a range of cell-based biotechnologies and facilitate hygiene control (e.g., polymers for hospital clothing, surgical equipment, *etc.*).

**Figure 3:**
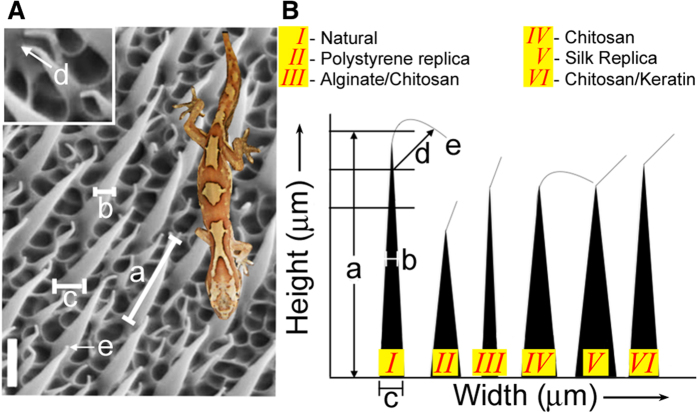
How we compared the effectiveness of duplicated spinules with natural examples. (**A**) Annotated SEM image depicting the distances between all of the main spinule surfaces, interfaces and separate spinules. These measurements were used to compare the efficiency and accuracy; (a) spinule height; (b) spinule width, midway; (c) spinule base width; (d) nanotip thickness; and (e) radius of curvature. A reference scale bar (bottom left of LHS image) = 1.5 μm. (**B**) A diagram comparing the shape and dimensions of an individual as well as a representative spinule constructed from culture plastic and biological materials that are typically used in experimental biomedicine and biotechnology, specifically chitosan, alginate, and silk fibroin. The width and height scales are not to true scale, but are comparative. It was assumed that spinule height is important for function, and ecologically, that assumption is correct regarding clearing dirt particles. However, wettability and bactericidal functions are nearly identical between all of the spinule compositions and the natural spinule. This diagram shows that the different material properties all replicate the shape of the spinules almost perfectly. However, the main differences relate to the spinule dimensions, particularly length, which does vary significantly between the spinule compositions. Another variance was the spinule tip curvature. (*I*) The natural spinule had a strongly inflected tip and thin base; (*II*) the polystyrene replica of the spinule had a large base and was less curved; (*III*) alginate and chitosan spinules were slightly thinner than the natural spinules and had a more pronounced tip curvature; (*IV*) chitosan-based spinules possessed a tip curvature identical to the natural spinules and almost the same height. (*V*) Similar properties were observed for silk. (*VI*) Spinules composed of chitosan and keratin were almost identical in height, with a bent tip and slightly less curvature than the natural spinule. In this diagram we have measured the tall spinules only. The short spinules in the packing arrangement are identical in shape, tip dimension and separation distance, but smaller in size by 3–5x.

We dissolved plastic petri dishes made from crystal grade polystyrene (TCP) in hexane and xylene, strong hydrocarbon solvents, to produce a polymer solution with a relatively high viscosity (honey-like). Over the course of “trial and error” experimentation with a range of low volume polymer solution concentrations that ranged between 2–8% w.v., we found that the length of the synthetic TCP spinules from SEM images (3.2 μm) (cast from the dorsal region of the gecko pelt) was 20% less than that of natural spinules at 4 μm (Fig. 2*ii, iii, v, vi*). This reduction in the accuracy of duplication was corrected by increasing the concentration of polystyrene solution to 9.5%, which produced a working viscosity 0.4 P.s. The average dimensions of the spinules at different angles and planes (as represented in Fig. 3) were remarkably similar to the natural microspinules. A large increase in the viscosity of the polystyrene solutions (25.8% w.v. polystyrene) however, eventually led to duplications with stunted lengths of spinules by as much as 10% (Supplementary Fig. S2).

Semi-viscous polystyrene solutions, ranging between 0.4–0.8 P.s., possessed fluid flow properties that allowed the solutions to permeate and infiltrate pore spaces between 1 μm and 50 nm that were embedded inside the negative mold to create a material devoid of trapped air pockets. The volume and density of the polymer, also ensured that it remained in the spaces throughout the drying and hardening processes that followed. With low viscosity polymer solutions below the <0.4 P.s. threshold level, misshapen structures and large holes in the patterned membrane resulted, due to trapped air and inadequate flow into all of the free spaces (Supplementary Fig. S3). We determined the threshold value by producing a series of polystyrene solutions with incremental increases in polystyrene volume. For each one of these we measured the viscosity and examined the replication result by SEM for quality. The solution was the first one in the series to generate accurate good quality microspinules with right curved hair shapes and dimensions as detailed in Table 1.

There was a reaction between the vinylpolysiloxane and xylene solvent soon after the polystyrene solution was applied, which led to a slight shape warping in the PVS material. This warping effect is not considerable but for the copy process to be best, the mold needs to be flat. To prevent this distortion, the PVS template was secured flat on each side with metal clips (Fig. 1 [step 5]). The polymer solution covering the mold surface was air-dried over night to facilitate re-setting of the sigma (C-C) bonds between the styrene monomer units and to completely remove the permeated solvent.

Select naturally derived solvents from botanic oils are less harmful than chemical solvents^31^. Although antimicrobial in their action, botanical solvents are easily dissolved away in water and are non-toxic in the concentrations used. Double-steamed distilled pine needle oil (50 to 97 percent monoterpene hydrocarbons dominated by a-terpinol, from *Pinus spp.*) or limonene (cyclic terpene from the rind of citrus fruits, e.g. grapefruit, lemon, bergamot, lime and orange etc.) were used as environmentally benign replacement for xylene.

Pine needle oil completely dissolved in the crystalline polystyrene to yield a transparent, colorless solution with a median viscosity of 0.6 P.s. and similar behavior to the xylene dissolved polystyrene that infiltrated the pore architecture inside the negative mold. Again, the pores remain filled with the pine-oil-dissolved polystyrene solution after the dry curing process.

### Qualitative analysis of duplicated spinule arrays

The siloxane mold possessed a surface energy at the boundary layer, which made it easy to remove the overlying polystyrene membrane with a pair of forceps (Supplementary Fig. S4). The reconstituted styrene membranes displayed high fidelity replicas of the spinule architectures at a nanoscale resolution with matching features in near-perfect arrangements. Comparing SEM images between the synthetic replicas and natural shed gecko skin, we observed bordering on perfect duplication of the microscales and honeycomb-based base structure. The spinules were packed together in exactly matched densities and configurations that fell into a hexagonal pattern. At higher magnifications, we observed an excellent replication of the spinules, with their high aspect ratios supported by splayed bases (Fig. 2A top two rows).

In addition to this electron contrast image, plane polarized light microscopy (Fig. 2B(*i*)) and enhanced halo free illumination of the gecko skin with Differential Interference Contrast (DIC) (Nomarski interference phase contrast) (Fig. 2B(*ii*)) confirmed the one-to-one physical match between natural and bioreplicated arrays of gecko skin spinules. Under normal brightfield white light microscopy, the geometrical order of the panels in the scales and the regular hexagonal arrangement of the spinules were instantly recognized by birefringence, which was multi-colored at the surface of every observed natural and replicated scale.

### Functional analysis of spinule arrays

Our method produced very good spinule copies. However, the most important objective of the study was to determine whether the non-wetting and anti-bacterial behaviour of the original was preserved. There were noticeable nanometer differences (between 100–300 nm) in the height, as well as spacing and curvature (Table 1) (possibly related to substrate elasticity) of the replica spinules. The anti-wetting (Fig. 2B(*iii* and *iv*)) and bactericidal properties however were not proportionally diminished (Supplementary Figs S5 and S6). The contact angle of water droplets on the replicas was consistently measured at 134 degrees compared to ≥136 degrees for the natural shed skin. Bacteria cultured on gecko replicas and natural skin were ruptured and extensively destroyed by the spinule nanotips Fig. 2B (*v* and *vi*)). This contrasted dramatically with healthy bacteria colonies, stained green for viability, existing on smooth polystyrene surfaces, where all the rod shape bacteria can be clearly observed (Supplementary Fig. S6D).

### Duplication of spinule arrays with biopolymers

**Figure 4:**
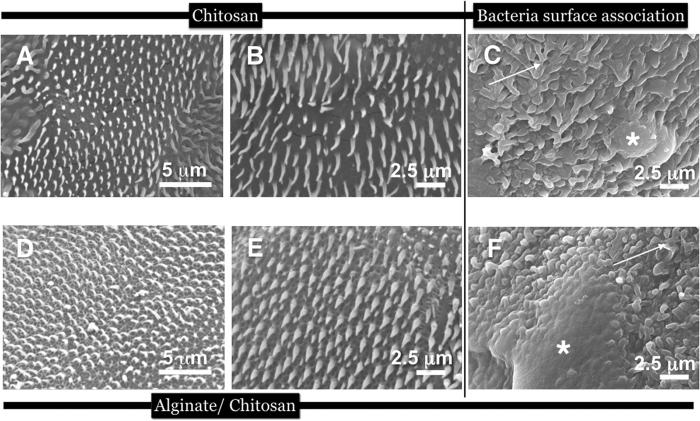
SEM imagery of replicas made with chitosan (5% w/v) and chitosan (5% w/v) and alginate (2% w/v) in 2 different aspects and magnifications, as well as attempts by living *Lactobacillus* bacteria to colonise the spinulated surface. Infact, the bacteria are shown to be eviscertaed leaving only smeared deposits (white asterix) covering the spinules (white arrow); In (**A**) the chitosan spinules were curved naturally. Furthermore, the spinules were softer and more flexible than polystyrene duplicates; (**B**) a higher resolution SEM image of the chitosan spinules in an array to focus on the shape of spinules more clearly; (**C**) Bacteria smeared over the spinules following rupture by the tips; (**D,E**) SEM imagery of replicas made with a bilayer of chitosan (5% w/v) and alginate (2% w/v) polyelectrolytes. The basal layer is formed by neutralized chitosan and the spinule protuberances were formed by calcium crosslinked (gelled) alginate (the reverse was also carried out with identical results). The curled tips formed after *in-situ* gelling of the biopolymer substrates. In contrast to the polystyrene spinules, spinules made from polysaccharide materials (in a dehydrated state) matched the tip curvature of native spinules more closely; (**F**) Bacteria grown on top of alginate-made spinules after 7 days led to more ruptured bacteria detritus (white asterix) covering the spinules (white arrows).

**Figure 5:**
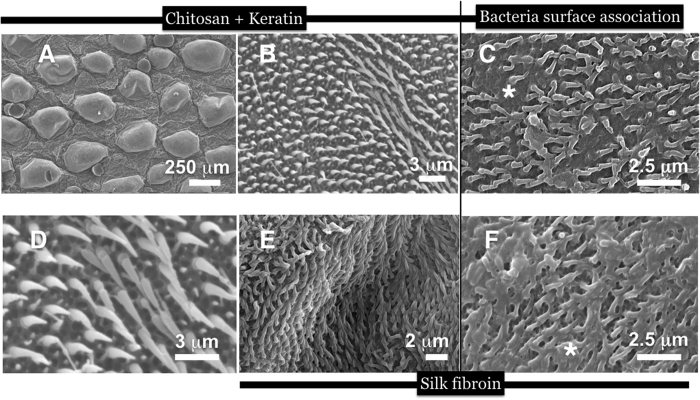
SEM images at the surface of a chitosan-keratin-blended gecko skin surface duplicate (*S. williamsii*) at 2 magnifications for representing the microstructures (the scales and the main region of the spinule) as well as the nanostructures represented by the nanotips and spinule bases. (**A**) A low magnification SEM image to show broadly the accurate copying of the gecko skin scales (the microstructure of gecko skin surface); (**B**) A higher power view of spinules at the top of the scale; (**C**) Bacteria cell rupture and death on top of chitosan/keratin blend microspinules (white asterix marks the bacteria detritus); (**D**) a magnified view of long spinules in the ridge seen in panel B; (**E**) A top-down view of silk fibroin spinules. The silk substrate led to good duplication of spinules in terms of matching structure and morphology. However, a large number coalesced due to the capillary forces on drying and collapsed horizontally onto the surface. Despite this phenomenon, extensive bacteria cell (highlighted) rupturing occurred depositing a large amount of detritus over the spinules, in a week, as observed in (**F**).

Confident that the method yielded excellent copies with proper functions, we produced replicas using a handful of biomaterials. We measured the hair dimensions, spacing between spinules (of height plus width at midpoint and the tip), periodicity and packing density to compare accurate spinule copies composed of the different biomaterials (Fig. 3). Spinules of the various replicas, closely matched the natural spinules in height and thickness. An exact match was found between natural versus replicas when the separation distances between the spinules were measured (Table 1 and Fig. 3A). When we compared the spinule morphology and dimensions between the different biomaterials, there were specific variations in height and base thicknesses. Small variations were observed in the nanotip curvature between the biomaterial and natural spinules. The biomaterials selected for this comparison and ultimate use in biotechnologies were chitosan (Fig. 4A,B,C), fused bilayers of chitosan and alginate polysaccharides (Fig. 4D,E,F), blended α-keratin hair extract (Fig. 5A–D), silk fibroin (Fig. 5E,F). Chitosan-constituted spinules generated structures that most closely matched natural spinules in terms of height, tip curvature and thickness (Table 1). These soft biomaterials displayed spinules that had a natural curvature that was not seen in the stiffer, crystalline polystyrene spinules. This result may be due to the rapid and more complete hardening of the material and filling the pore network due to the permeation of the cross-linking agents. Polystyrene spinules were hardened differently by slow evaporation of the organic solvent. However, during the curing process, the silk fibroin spinules often adhered to each other and clustered together due to capillary forces that occurred during the drying process. They were also seen to collapse horizontally onto the surface (Fig. 5E). After 7 days of culture, bacteria covered the tops of the spinule arrays. We calculated a high percentage of bacteria (95%) that were destroyed on polystyrene and biopolymer duplicates (as shown by SEMs of the melted detritus from bacteria) due to interaction with the topography through a stochastic combination of stretching between spinules and nanotip piercing (Figs 4C,F and 5C,F; Supplementary Figs S5 and S6). The differences in spinule dimensions between the different biomaterials, however may affect the responses of eukaryotic cells.

Our method may also be utilized for skin attachment on a surface, which does not represent a flat adhesion plane. For example, instead of a glass silica slide, the surface can be a glass rod (cylinder), which more closely reflects the original macro shape of the organism (lizard body). In addition, the interacting liquid and adhesion surface can be chosen (and tuned with polarity) according to the interfacial skin requirements, resulting in an unraveled fixed sample on the surface. In the case of many gecko species, the upper skin surface is typically hydrophobic (CA 90–150°)/superhydrophobic (CA > 150°) and the underside is hydrophilic. Thus, a liquid and surface with opposite polarity (water and silica) provides a contrast for replica preparation (the numerous species studied by the authors included *Amalosia* sp., *Diplodactylus* sp., etc.).

## Discussion

Biotemplating (bioreplication) is a translation strategy for duplicating accurate copies of small biological molecules, objects and structures on biological surfaces, such as bacterial proteins, viruses, insect eyes, butterfly wings and pollen grains^21^,^25^,^32^. Soft lithography, which is used in the semi-conductor industry, is an alternative, precision microfabrication technique that can be used to faithfully duplicate small biological structures. New chemistry-based lithographic strategies have increased the resolution power and automation, giving these strategies a superior advantage over casting and molding. One such novel technique (called STEPS) involves the electrodeposition of conductive polymers onto Bosch etched silicon wafers. This approach has yielded impressive high aspect ratio micro/nanoprotrusions in arrays with nature-emulating “bent and tapered” morphologies. The structures made with this method are heterogeneous and the edges, interfaces and joints are poorly delineated from the smooth edges and gradients of materials in naturally derived nano-constructs.

One of the simplest biotemplating techniques is “*casting and molding*”. To facilitate the smooth translation between nature and technology, it is important to manufacture a fixed, permanent and reusable negative master. Metals are an excellent choice for use as a master because of their physical strength; however, silica delivers equivalent toughness and resilience for the purpose of preserving both the shape and integrity of the open-space between spinules. Surface protrusions, which coexist at microscopic and the nanoscopic scales, present a fabrication challenge for casting materials in tiny spaces and preserving the shape of the mold. High aspect ratio hair-like structures, which are common throughout biological surface systems, are particularly difficult to emulate because they are largely intricate, display fine detail and are patterned in complex arrangements. In this study, we used a robust, inexpensive polyvinylsiloxane (PVS) impression material as the enduring and frequent use negative template.

The gecko spinule array was evolved for multi-functional purposes including removal of dirt and foreign particles from the skin surface without any chemical and biological support. Lipids and proteins exist on the surface of gecko skins, but these chemical components require topographical augmentation to enhance some properties such as hydrophobicity responses. The reported discovery that the spinules carpeting the surface can destroy living bacterial cells on first contact and deter bacterial biofilm organization via stretching, piercing, superconfinement and scrambling the structural information of bacterial cells that are used for attachment and multi-cellular packing arrangements represents a unique design principle for bactericidal surface technologies. One area of interest is the gecko patterning (by stenography) of biofilm-prone healthcare materials, which is of considerable interest due to the powerful emergence of antibiotic resistance. Antibacterial and anti-biofilm surfaces based on biophysical principles may prevent the microevolution of bacterial resistance.

This simple action of duplicating a surface of gecko spinules at sufficient resolution is necessary to simulate the natural self-drying and self-cleaning adaptations of gecko skin within environments that correspond to the physics of natural gecko habitats. There are no qualified observations from studies of their natural history inside of their natural habitat that showed that spinules actively destroyed bacterial cells and other microscopic organisms, which could only be affected by these small structures. Additionally, we cannot be sure that this is an evolved strategy for fighting microbial colonization in conjunction with the super-physical properties.

The self-cleaning mechanism with rolling water droplets is a strategy for removing contaminants and, presumably, small organisms. Therefore, direct replication of the gecko structure may not be necessary. For instance, we found that bacterial killing occurred on replica spinule structures that were smaller in height than those of natural gecko skin. Provided that the spacing of spinules is smaller, or comparable to the width of the bacterial cell, then the rupturing process will occur where the bacteria initially orient themselves on top of the spinules, presumably from stretching. We have observed 4 separate potential rupturing mechanisms among the nanotipped spinules, including compression (potentially causing local stretching around spinules), stretching, tearing and piercing where bacteria (depending on size) interact with the spinules from the tops or alternatively between them if space permits (smaller bacteria).

The manner in which bacterial cells behaved within the constrained physical spaces between spinules was imaged using confocal microscopy and showed cells in various patterns of arrangement; in parallel with spinules, vertically or horizontally straddling between them (Supplementary Figs S5 and S6). For comparison, bacteria interacting with biopolymer smooth surfaces are shown in Supplementary Fig. S8. Cells lying horizontally with spinule tips along the perimeter of the cell were particularly prominent. Additionally, bacterial cells were frequently observed in vertical alignment, which disrupted normal cell organization and biofilm formation. This method, while not innovative by itself, for the first time has been shown to work on small structures straddled between the micro/nanoscale made from a small highly selected sample of medically relevant biomaterials.

We were able to replicate these gecko micro/nanostructures using crystal grade polystyrene and prominent/popular natural biopolymers, including silk fibroin, chitosan, alginate and human hair keratin. Demonstration of spinule duplication with these materials was significant because they possess substantive clinical potential for tissue engineering (cell scaffolds) and wound healing (bandages). Successfully managing biomimetic translation does not require exact copies of the original. The pivotal idea behind biomimetics is that under scrutiny, important elements should give rise to a functional strategy. This principle seems to hold true with regard to bactericidal efficacy since spinule duplicates with shortened lengths and a less pronounced curvature compared to the natural version, lead to identical bactericidal properties. We discovered that the key functional design features were the existence of nanotips, the spinule spacing and possibly the flexibility of the spinules. However, it is still extremely important to copy the structures within certain limits. Further shortening of the spinules gradually increases wettability and reduces the self-cleaning capacity of the spinule arrays.

As we have observed in this study and as shown by other studies, various bacterial cells cultured on synthetic gecko spinules were mechanically constrained in their arrangements and rupture occurred, particularly due to puncture by the nanotips. According to Hochsbaum *et al*. the nanopost disposition, pitch, density and flexibility of the posts varied the responses of bacteria grown on such arrangements^33^. Therefore, in future, we will focus on how to exert more control of the responses by using different species of gecko and different regions of the body as well as relating their natural history and evolutionary adaptation to the skin structures. Because there is significant variation in size and distribution between individual species, their body regions correlate to their physical habitats. This approach may lead to more adjustable levels of biofilm control, bacterial selectivity and cell rupturing where select species of gecko may exhibit certain enhanced functional properties based on their behavior, habitat and environmental conditions. We may be able to identify, select and duplicate a design that caters to killing particular bacterial strains and species that vary in motility and susceptibility to mechanical damage.

In addition to applicability to geckos (the largest group of lizards, with over 1000 species, some of which are rare and/or endangered), the protocol that we described in this study may also have the potential to replicate exterior nanoprotuberances in other organisms that molt or have delicate epidermal layers. Finally, the end use for these replicas will eventually include, but will not be limited to, supersurfaces for anti-microbial, self-cleaning and eukaryotic cell modulation, which are based on expanding the vitality of nanotopography influences on cell behavior.

The bioreplication method developed in this study will enhance efforts to exploit gecko skin spinules as an antibiofouling device. It will facilitate the proper testing and evaluation of the potential of spinules to fight disease-causing bacteria. The next stage in the evolution of this biomimetic transfer is to expand the gecko-patterned membranes into more usable panels of material with large surface areas. One feasible method would be of computer-controlled lithography through microcontact-printing, in which a PVS stamp, which we used for biotemplating, is molded with gecko skin spinules. The patterned stamp would be forcibly applied onto a soft substrate to transfer the spinule shapes into newly prepared material. Moreover, it is now feasible to print the structures with the help of 3D imaging techniques (X-Ray microscopy and 3D CLSM) to provide the necessary digital datasets (Supplementary Fig. S7).

## Materials and Methods

Materials included: gecko skin moults e.g., *S. williamsii* and *Lucasium sp*. (hand captured and reared in NE Queensland); Take 1 Advanced RB wash SF (Kerr Dental Corporation); SPL Life sciences crystal grade polystyrene petri dish (430167-Dow Corning); natural Pine needle oil (Sigma W290500); hexane and xylene (Extra pure grade, Duksan Reagents); calcium chloride (Sigma 93% granular); sodium hydroxide (Sigma ACS reagent >97% 221465); chitosan powder (5 MW: 90DD) Heppe Medical Chitosan GmbH) sodium alginate powder (FMC Pharmaceutical grade Protanal CR 8223); Urea (Bio basic Canada ultrapure >99.5%, UB0148); Thiourea (Sigma ACS reagent T8656 > 99%) 2-mercaptethanol, Sodium carbonate (Sigma 99%), Lithium bromide (Daejung Chemicals), Cell tracker deep red dye (Thermo-Fisher Scientific).

### Shed Gecko skins

Intact shed gecko skins were utilized in these studies (Australian spp.). Geckos (*Lucasium sp*. and *S. williamsii*) were captured at night by hand from North Eastern Queensland (QLD). Only healthy adult lizards were returned to the laboratory with a heat source for thermoregulation and suitable plant foliage and water. They were fed domestic European crickets (*Acheta domestica*) three times a week. Geckos were allowed to shed twice, with no human handling before skin collection. This work was conducted under Ethics Approval A1676, and QNP permit WITK05209908. The experimental protocols were approved by James Cook University, Townsville, Australia. All methods related to use of live geckos were carried out in strict accordance with all guidelines for animal husbandry. No geckos were harmed in this experiment.

### Preparation of biopolymer and polystyrene solutions

#### Polystyrene

Polystyrene petri dishes (430167-Dow Corning) were cut with scissors into small fragments. The fragments of Crystal grade TCP cut segments were dissolved in 100 mL Xylene (Duksan Reagents extra pure grade) or pine needle oil for 12 hours to ensure complete dissolution. The viscosity of the prepared solution was calculated according to the equation: viscosity = [2(ps − pl) ga^2^]/9 v, from measurements recorded from a standard “dropping ball” method.

#### Chitosan

This material extract from crab shells was formed into a solution by a conventional method^34^. Pharmaceutical grade (5 MW: 90 DD) Heppe Medical Chitosan GmbH was dissolved in distilled water (10 g in 200 mL) containing 2% acetic acid and mixed thoroughly for 1 hour.

#### Silk Fibroin

Silk fibroin was extracted from dried silkworm cocoons according to the published method of Rockwood *et al*.^35^. In brief, 5 g of cut fragments of silkworm cocoons were soaked in a boiling sodium carbonate solution. Following washing and drying of the remaining silk fibroin threads the material was dissolved in an aqueous solution of lithium bromide (9 M) for 4 days at 60 °C. The solution was dialysed against distilled water for a further 4 days.

#### Alginate

FMC Pharmaceutical grade Protanal CR 8223, sodium alginate (Lot: GQ9800602) (2 g in 200 mL) was dissolved in sterilized distilled water and mixed thoroughly with a magnetic stirrer for 2 hours at room temperature. Gelation of the alginate was carried out directly on the mold through mixing a 100 mM calcium chloride solution onto the alginate layer deposit.

#### α hair keratin

Extracts were prepared according to the method of Nakamura *et al*.^36^. Human hair, cut away from the primary author (10 g), was treated with hexane to remove lipids from the hair surface. The removal and use of human hair was undertaken in accordance with guidelines and regulations for human subjects in experiments, but due to the non-invasive nature of the procedure it did not require ethical assessment and approval. Neither did it necessitate informed consent. All methods in this regard were approved by Yonsei University. The lipid free hair (50 mg) was cut in to fine pieces with scissors followed by finer cutting with a scalpel blade. The hair was immersed in a solution containing 25 mM Tris–HCl, pH 8.5, 2.6 M thiourea, 5 M urea and 5% 2-mercaptoethanol (2-ME) (Shindai method). The dissolved keratin solution was filtered and centrifuged for 20 minutes at 15000 × g. In the final step, keratin solution was dialysed against pure distilled water for 4 days with daily replenishment of distilled water^36^.

#### Biopolymer blends

Mixtures of chitosan and αkeratin were prepared. αkeratin (2%) was added into a solution of chitosan (5%) in a 50:50 ratio by w/v, and thoroughly mixed before applying into the mold.

### Presentation of shed Gecko skins for casting

The fragile shed gecko skin can be spread uniformly onto a glass slide and presented for the first casting step. A film of water is applied to a single glass slide. The gecko skin is then placed onto the film. The surface carpeted with spinules must face upward so that the casting substrate could be applied to the spinules. The skin segment was gently placed onto the film of water. Immediately afterwards the entire hydrophilic (CA = <21 degrees) underside was attracted to the water. The spinule-coated side is hydrophobic (CA = >134 degrees) and is repelled by the water. The gecko skin is also highly elastic (partly due to its small thickness) and the product of these phenomena is to instantaneously propel the skin into a panel in the correct orientation for casting (Supplementary video 1). The skin is dried in an oven at 60 °C for 30 minutes to evaporate the water film and dry bonding of the skin onto the glass surface.

### Casting of the negative mold

A proprietary siloxane based dental impression material (Take 1 Advanced RB wash SF) was applied to the surface of the flat panel gecko skin, already pre-fixed onto a glass slide. The substrate hardens within 30 seconds after dispensing and seals itself deep between the 500 nm separation (in x, y planes) space of spinules and into the voids within the surface honeycomb structure. The cast was peeled away cleanly from the skin (the skin can sometimes be re-used) and inverted in readiness for casting with the polymer or biopolymer solution. Viscosity of the solution is critical for accurate replication. Polystyrene solution with a viscosity between 0.4 P.s and 1.5 P.s produced copies of the spinules, en masse, with 90% fidelity.

### Casting of the positive mold

The polystyrene (crystal grade melt) or chitosan (5 MW: 90 DD Heppe Medical Chitosan GmbH) or alginate (FMC Pharmaceutical grade Protanal CR 8223) solution (and their various blends) was pipetted and soaked into the molded pattern of pores. As a guide, 1 mL of polymer solution is dispensed onto the negative mold measuring, on average for the *S. williamsii* and *Lucasium* molts: (*l*) 4.5–6mm × (*w*) 2.5–3 mm. In this way after drying, a solid membrane is generated with a thickness of 2 μm–5 μm. The polymer-coated mold is left to air-dry overnight inside a running fume hood. The casted polystyrene material was easily peeled away, manually with a pair of forceps. The chitosan-coated mold was neutralised with 5 M NaOH solution, washed onto the dried chitosan membrane, for 30 minutes until rigid and was then peeled away, manually with a pair of forceps. Alginate coated molds were cross-linked by adding a 100 M calcium chloride solution.

### Microscopy and Imaging of natural and synthetic gecko spinules

#### Scanning electron microscopy (SEM)

Gecko skin and replicas were imaged under SEM to show the micro-/nanoscaled spinules and accompanying architectural features at the surface. Samples of natural gecko skin and bacteria populated natural gecko skin and their replicas, were fixed in 4% PFA for 12 hours, then attached onto individual SEM stubs and air-dried for 2 hours. All samples were sputter coated in platinum, using a Hitachi E-1010 machine. Imaging was carried out using a Hitachi S-3000N Scanning Electron Microscope.

### Contrast Microscopy in Brightfield light

Gecko samples were viewed in bright light under a Leica compound microscope DM5500, in high contrast mode and also under plane polarised light. We used Differential Interference Contrast (DIC) (Nomarski interference phase contrast) to highlight the different patterning styles and periodic ordering of the spinules, which lead to iridescence effects.

### Laser confocal microscopy

Laser confocal 3D digitally rendered microscope images were taken of the natural gecko skin spinule array and polystyrene replicas to highlight similarities in spinule dimensions (height in microns, thickness at the base and the tip and spacing in nm), shape and morphology. Spinules were dyed with Cell Tracker (CT) Deep Red (Thermo-Fisher Scientific: ex = 630; em = 650) for 30 minutes in phosphate buffered saline (PBS), mounted onto a microscope slide then scanned 50 times at the highest magnification, without pixelated distortion, from the top of the spinule to the base (×1000 with ×1 digital zoom: pixel size = 0.38 μm; Zeiss Confocal Laser Microscope) to generate 3D composite images [Picasa Image settings used to better resolve the spinules into clear view were used in the following order: sepia, cross-process, HDRish, Picasa setting; Image dimensions 22 × 22 μm; B = 22 × 22 μm]. Of the fluorescent biodyes tested, deep red coated effectively on the natural and the polystyrene versions. Both green and red lasers were used on natural and replica samples. The technique was also used to image bacteria behaviour and associations with natural spinule and replica arrays. This multi-dimensional dataset, representing the spinule carpet in 3D, can be translated into digital data formats for 3D inkjet printing.

### Measurement of spinule characteristics and distributions

Digital SEM images of natural spinules and replica spinules were measured in different aspects using Image J software and compared with each other. The spinule length (or height in microns), between spinule distance (in nm), nanotip width (in nm), width of base (in microns) and spinule density per unit area were measured among natural spinules and in all other replicated spinules. The results were tabulated to compare and contrast the replicas between different biomaterials and crystal grade polystyrene.

### Functional evaluation of wettability between natural skin and replica

A single 25 μL drop of distilled water was dispensed from a 200 μL pipette tip onto the gecko skin surface and polystyrene replica. This size droplet is typical of ‘real’ droplet size encounters however it should be noted that for contact angle measurements it will be affected by gravity and will underestimate the contact angle (in comparison to droplets <10 μL)^37^. The sitting drop was imaged and photographed with a Surface Electro Optics apparatus. The static contact angles of the water droplet surface related to the horizontal are drawn onto a camera image fixed to a goniometer, for reference and measured instantaneously via image XP-5.6 analysis software (Surface Electro Optics SEO, Phoenix 300). A record is provided of left and right angles at the contact between drop and surface, wetting energy and spreading coefficient and work of adhesion for the examined drop.

### Functional evaluation of bacterial killing between natural skin and replica

A co-culture of 7 gut bacteria were prepared and cultivated on natural shed skin and replicas. *Lactobacillus* casei 3%, *Lactobacillus rhamnosus* HA-114: 10%, *Lactobacillus rhamnosus* HA-111: 25%, *Lactobacillus acidophilus*: 45%, *Lactobacillus salivarus*: 1%; Lactobacillus plantarum: 10%, *Bifidobacterium longum* 3%: 5%, *Lactobacillus plantarum*: 7% [stearic acid, silicon dioxide, ascorbic acid and potato starch]. One capsule of probiotic human bacteria strains (Udos blendTM consisting of 8 different bacterial strains) was added into 50 mL of plain DMEM media and cultured for 48 hours in anaerobic conditions at 37 °C. A single capsule was loaded with potentially 1.4 billion viable CFU bacterial cells. Samples were dropped into the culture broth and suspended there for 48 hours to 4 weeks. Sample membranes were removed washed and fixed in cold acetone prior to imaging under SEM.

## Additional Information

**How to cite this article**: Green, D. W. *et al*. High Quality Bioreplication of Intricate Nanostructures from a Fragile Gecko Skin Surface with Bactericidal Properties. *Sci. Rep.* **7**, 41023; doi: 10.1038/srep41023 (2017).

**Publisher's note:** Springer Nature remains neutral with regard to jurisdictional claims in published maps and institutional affiliations.

## Supplementary Material

Supplementary Material

Supplementary Video

## Figures and Tables

**Table 1:** The spinule dimensions and spacing were almost identical between natural shed gecko skin and polystyrene replicas (n = 4).

Spinule dimensions and geometry (dorsal surface)	Natural	Synthetic Replica	Synthetic BioReplicas	Chitosan	Silk fibroin	Chitosan/αhair keratin blend
	Natural shed gecko skin (Strophurus williamsii)	Polystyrene	Alginate/chitosan composite			
Spinule length/ height from base Fig. 3(a)	3.0 μm (±0.5 μm)	1.0–1.5 μm (±0.5 μm)	2 μm (±0.5 μm)	2 μm (±0.5 μm)	2 μm (±0.3 μm)	3.4 μm (±0.5 μm)
Spinule width (base) Fig. 3(c)	350–450 nm	350–400 nm	350–400 nm	350–450 nm	300–400 nm	350–450 nm
Spinule array spacing (tall and short)	500 nm	600 nm–1 μm	800 nm–1 μm	500 nm–1 μm	500 nm	500 nm–750 nm
Spinule density	8*/* μm^2^	8*/* μm^2^	4*/* μm^2^	8*/* μm^2^	8*/* μm^2^	8*/* μm^2^
Tip radius of curvature Fig. 3(d)	10–50 nm	200 nm	200 nm	10 nm	50–100 nm	50–100 nm
Spinule tip dimension Fig. 3(e)	50 nm	50–80 nm	50–60 nm	50 nm	50–85 nm	50 nm

The tip curvature varied considerably between the replicas (alginate/chitosan (Alg: 2% w/v and Chit: 5% w/v), silk fibroin (8%), chitosan (5% w/v) and chitosan/Keratin blend (Chit: 5% w/v and Ker: 1% w/v), which was related to the substrate fluid mechanics and the way that the substrate flowed and hardened during casting. The spinule length and packing distribution were more strongly correlated with anti-wetting, self-cleaning and anti-fouling effects. However, the correlation of the spinule length and bactericidal activity was not strong. The spinule density, nanotip size and curvature were characteristics directly related to the bacterial killing properties. We measured the length or height of primary spinules. Secondary spinules exist in the packing arrangement with identical tip width and shape of curvature but shorter heights and reduced dimensions (shaft width) by 70–75%. *The physical dimension categories are represented in Fig. 3 (the average measurements were calculated from a total of 20 randomly selected tall and short spinules across the sample surface).
