# Supplementary material for: High Quality Bioreplication of Intricate Nanostructures from a Fragile Gecko Skin Surface with Bactericidal Properties
Source: Sci Rep. 2017 Jan 25;7:41023. doi: 10.1038/srep41023 (PMC5264400; doi:10.1038/srep41023)
Supplement: Supplementary Material [file srep41023-s1.pdf]

High Quality Bioreplication of Intricate Nanostructures from a Fragile Gecko Skin Surface with Bactericidal properties

David William Green[1], Kenneth Ka-Ho Lee[2], Jolanta Anna Watson[3], Hyun-Yi Kim[1], Kyung Sik Yoon[1], Eun-Jung Kim[1], Jong-Min Lee[1], Gregory Shaun Watson[3*], Han-Sung Jung[1,4*]

Supplementary material

Figures

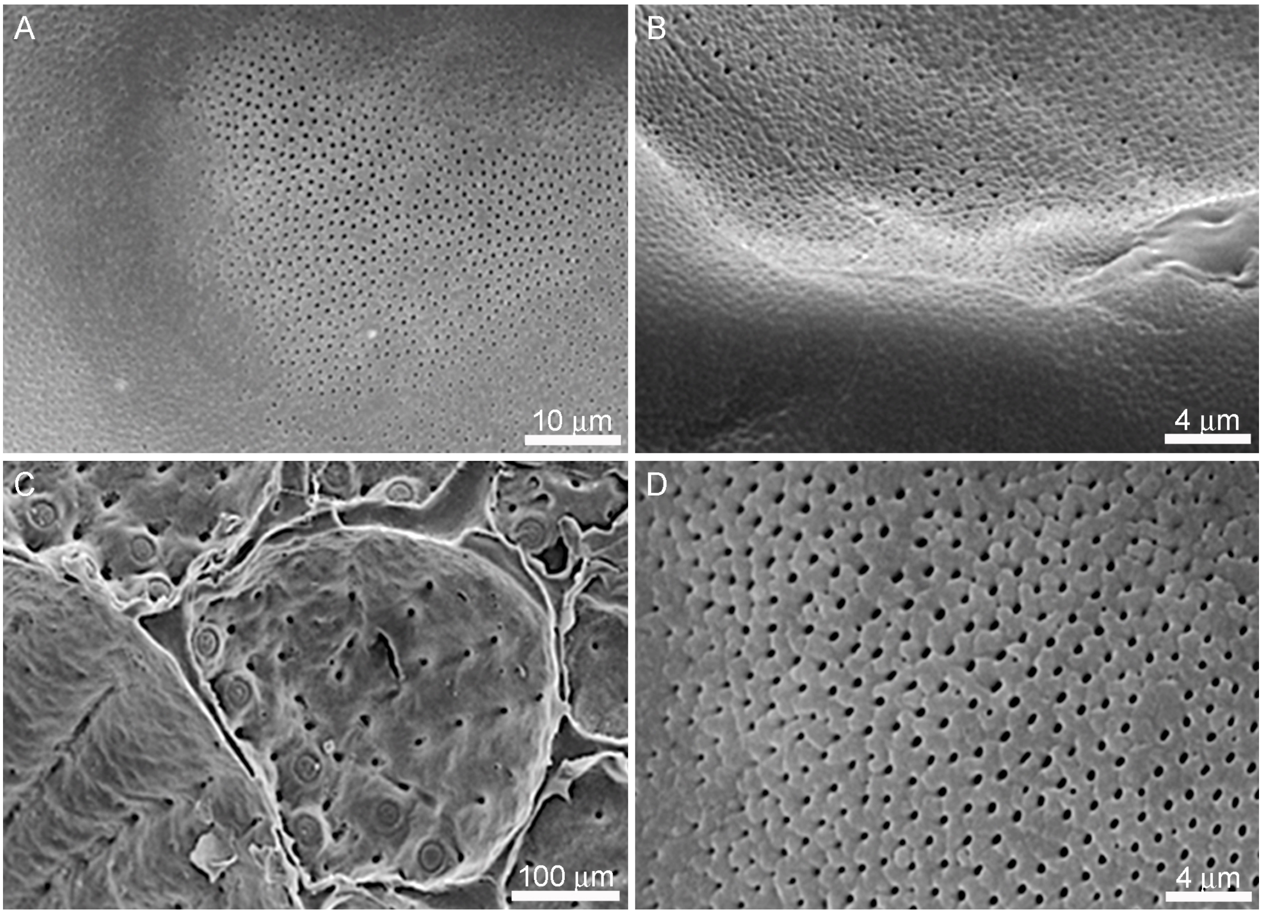

Fig. S1 SEM images of the surface of a negative imprint of shed gecko skin. (**A**) A surface view at low power and (**B**) surface view at higher magnification. The mold is made with a fast acting polyvinylsiloxane, Kerr's dental impression material. The pits and grooves are where the spinules and folds existed during casting. The geometric pattern of the spinule array has been preserved. (**C**, **D**) SEM images, at low and high resolution, of the negative vinylsiloxane pattern from the shed skin of *Nephurus wheeleri*. Thus, the procedure is applicable to other shed lizard skin surfaces, replicating other structures and features.

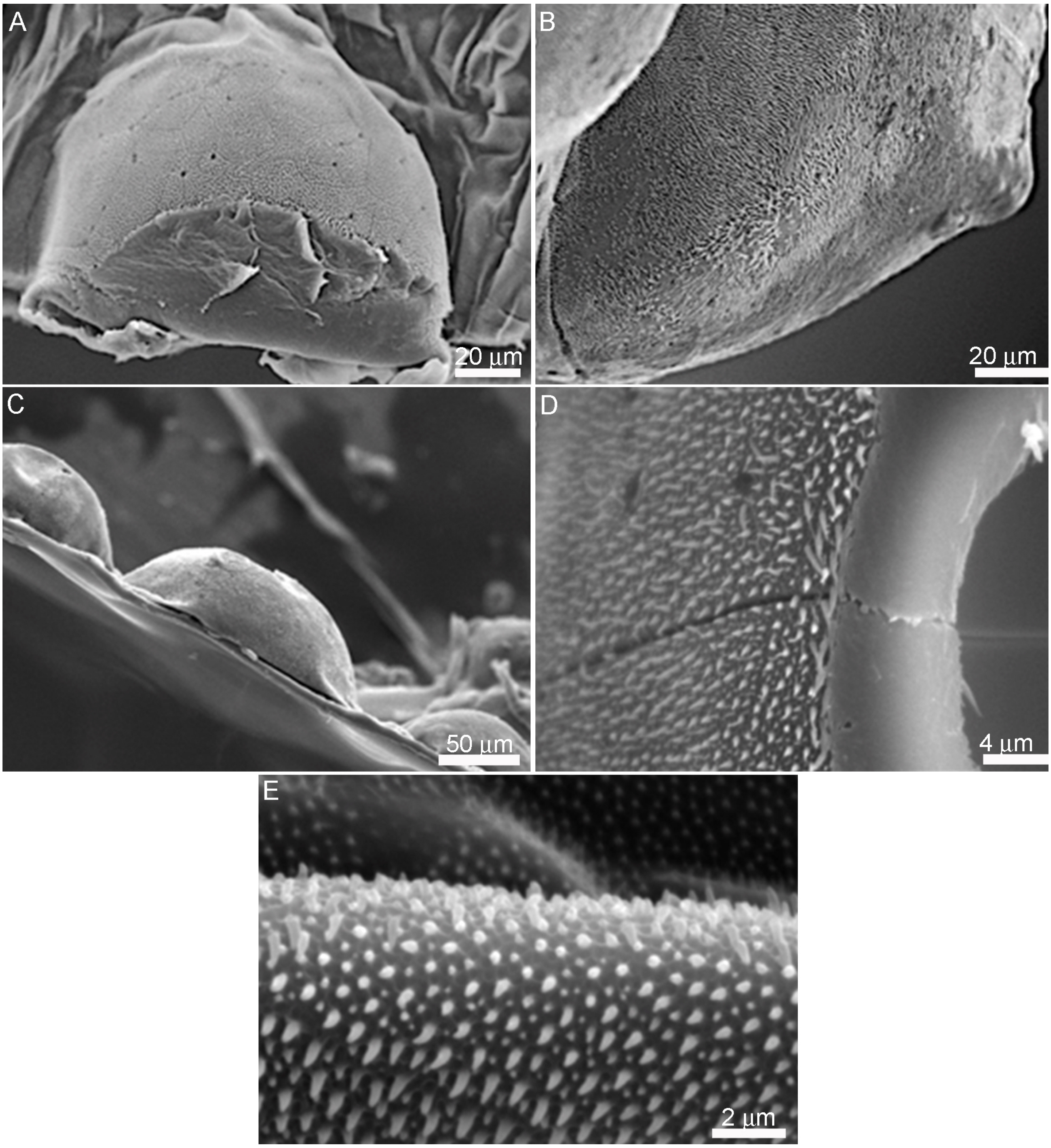

Fig. S2 SEM views of copied single gecko skin scale (*Lucasium sp.)*. In (**A**) the scale has been bisected. The microstructure of the scale and the small spinule nanostructure has been faithfully replicated. (**B**) High power examination at the surface of the single scale to show the replicated spinules. (**C**) An SEM cross-sectional view of the positive polystyrene copy of the scale microstructures. In (**D**) A cross-section through the gecko lizard patterned film copy, measuring 7 µm thick. (**E**) Reveals a positive copy of the gecko lizard skin, revealing surface ornamentation over complex topologies such as, folds derived from the original gecko skin.

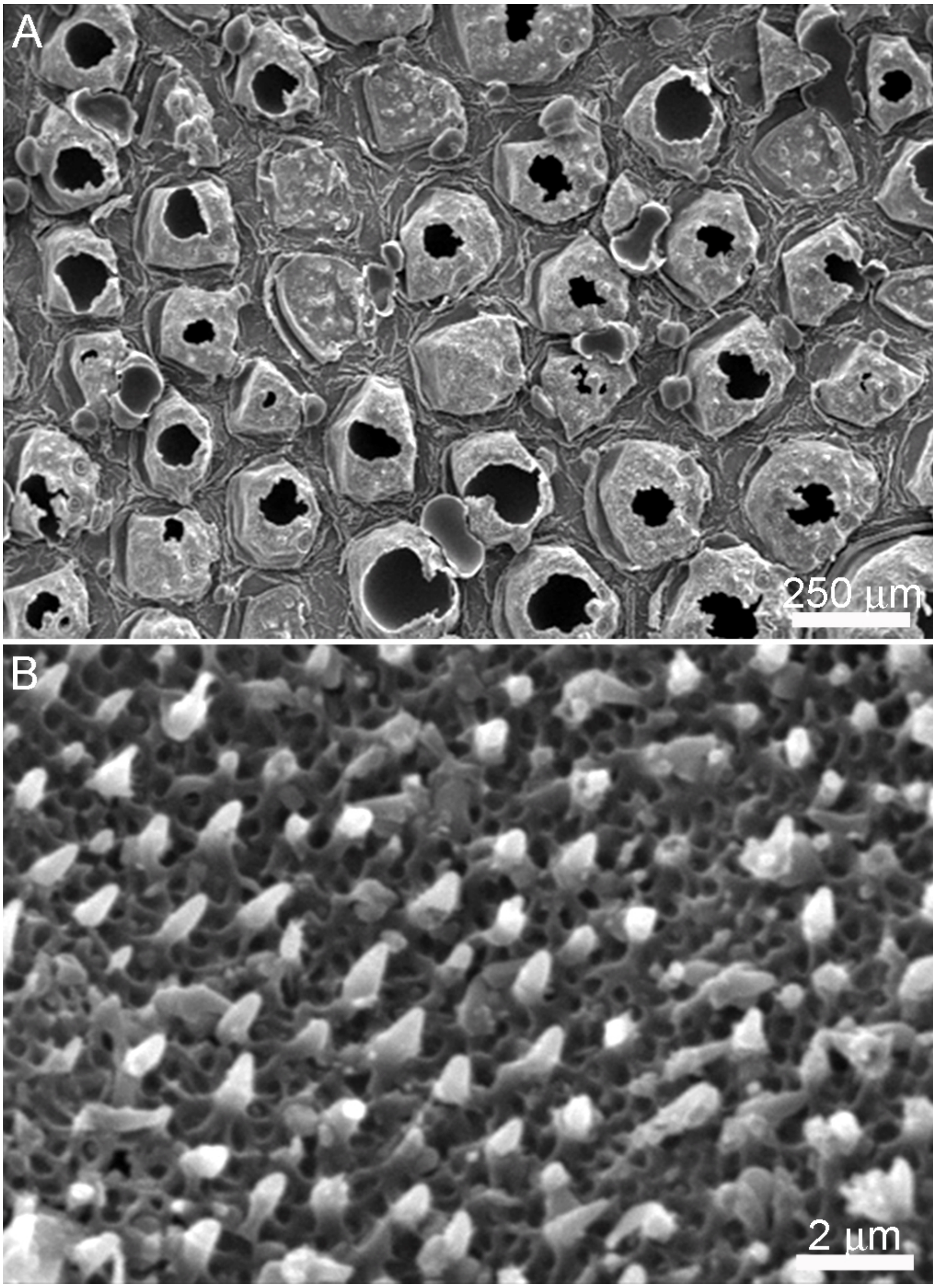

Fig. S3 (**A**) A positive replica made with a low viscosity polystyrene liquid (< 0.4Pa threshold). In this example, the scales have not formed properly leaving holes at the top part of the scale. (**B**) In the same sample, the spinules are stunted and malformed.

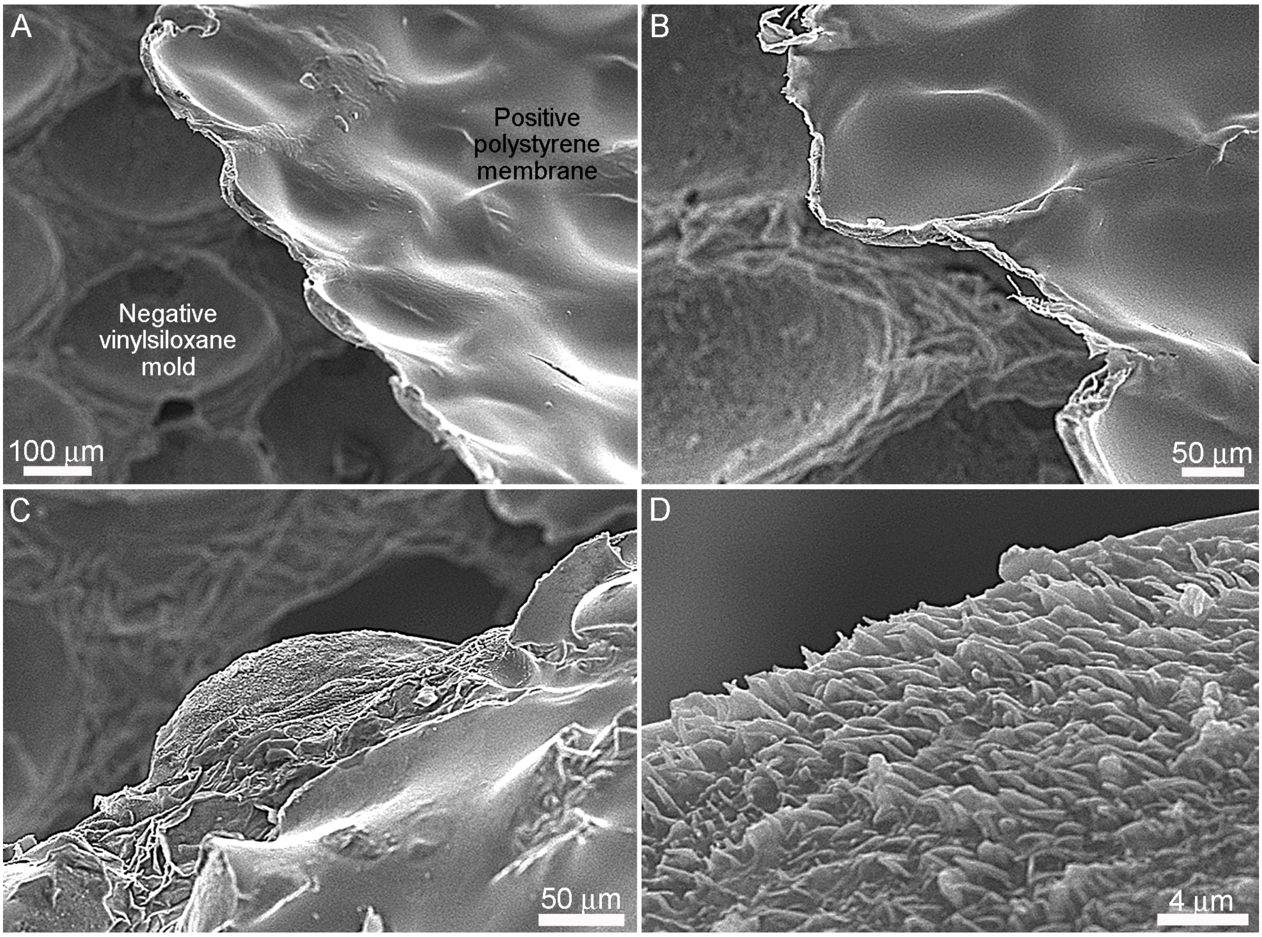

Fig. S4 SEM images relating to the attachment to and removal from of the polystyrene membrane from the negative vinylsiloxane mold (**A**) A section of polystyrene that has been peeled away from the PVS mold; (**B**) A higher magnified view of the peeled polystyrene membrane; (**C**) A view of the peeled polystyrene surface that was molded into the negative mold to show the roughness of the spinule bearing surface; (**D**) A highly magnified view of the molded polystyrene surface to show the dense coverage of spinules present there.

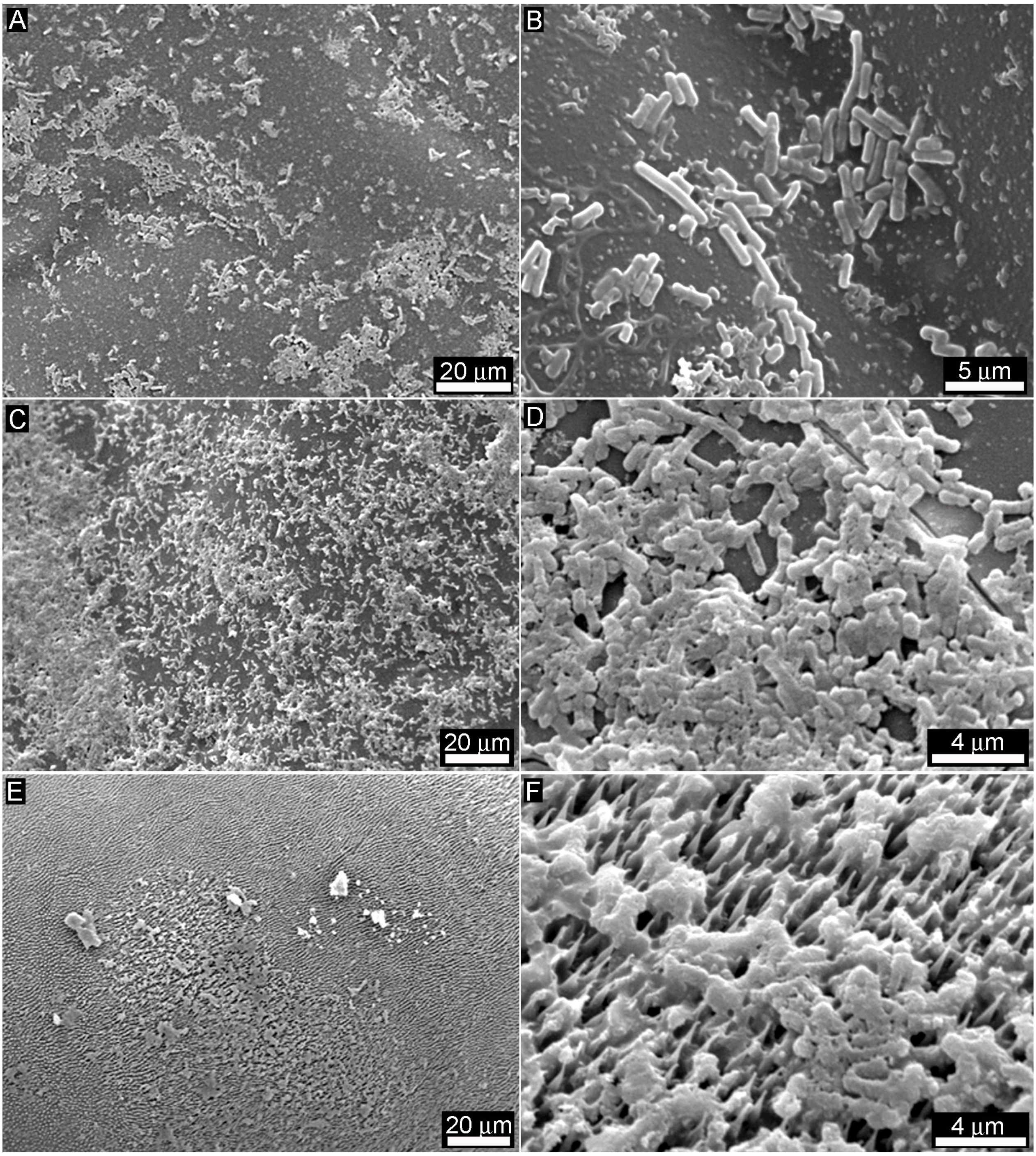

Fig. S5 SEM images highlighting the shredding and rupture of bacterial cells by the replicated gecko spinule array. Bacterial cells included *Lactobacillus casei 3%*, *Lactobacillus rhamnosus HA-114*: 10%, *Lactobacillus rhamnosus HA-111:* 25%, *Lactobacillus acidophilus*: 45%, *Lactobacillus salivarus: 1%; Lactobacillus plantarum*: 10%, *Bifidobacterium longum 3%:* 5%, *Lactobacillus plantarum*: 7% [stearic acid, silicon dioxide] on the smooth, reverse side of the (**A**) natural gecko shed skin (**B**) the skin replica. (**C**, **D**) The colony forming units of *Lactobacillus* bacteria growing on a smooth chitosan membrane surface, without spinules, after 7 days. In comparison to the

spinule coated surface bacterial cells are intact and generate large dense colonies that cover 90% of the free surface; (**C**) An SEM low power view of bacterial cells and biofilm formation on the smooth surface; (**D**) A SEM high power view of the bacterial cells attached to the smooth chitosan surface. (**E**, **F**) Showing bacterial growth on natural shed gecko skins after 7 days. The loading of bacteria was exceptionally high, roughly 1 billion. (**E**) An SEM low power view of bacterial cells on the natural gecko skin; (**F**) An SEM high power view of bacterial cells on top of the natural gecko skin spinules to show bacteria lysed cells *en masse*. The density of bacterial cells is noticeably lower than the smooth control surfaces.

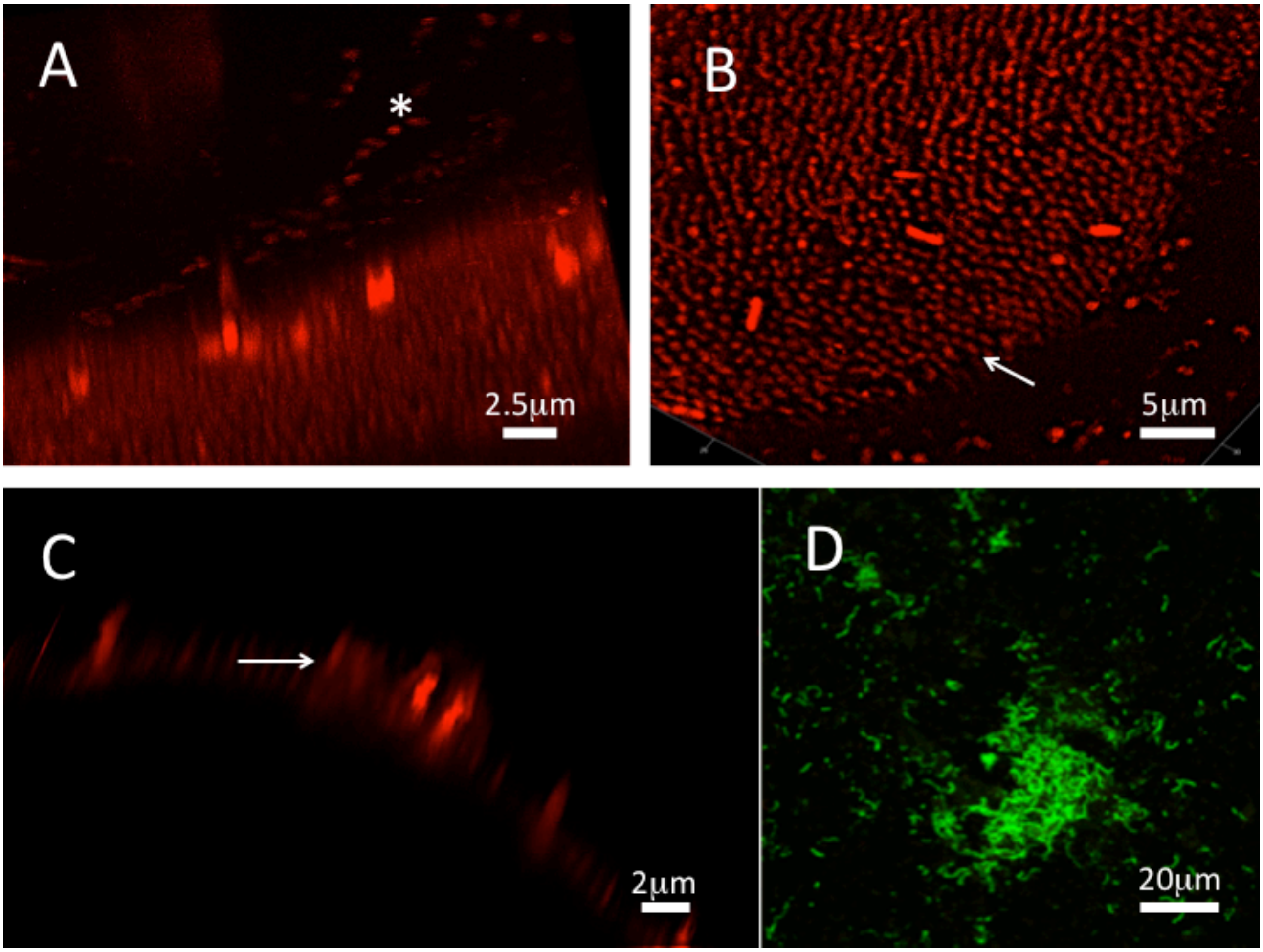

Fig. S6 Laser Confocal Imagery of TO-PRO-3 fluorescently stained Lactobacillus bacteria at the surface of polystyrene replicas in different plane views and magnifications (A-C) to highlight the arrangement on top of, and between the spinules. The deep red TO-PRO-3 stain fluoresces within dead bacteria having a damaged cell wall. The bacteria have been cultured for 7 days at a high billion-scale density, on the polystyrene (PS) surface. This was

identical to the number loaded on similar samples displayed in the previous SEM images. The spinules also absorb the fluorescent dye and show-up red; (A) A edge-on view of synthetic spinules some of which are covered in bacteria (strong red). The asterix marks the floating bacteria that fall to the surface in seconds (scale bar= 3 μm); (B) A top-down surface view to show individual cells interacting with the spinule array (Arrow points to the tops of the spinule array on a single scale) scale bar= 5 μm); (C) The synthetic spinule coated membrane viewed side-on showing two single bacteria aligned parallel with the spinule (scale bar= 3 μm); (D) A smooth polysyrene surface cultured with bacteria for 7 days, this time stained with TO-PRO-3 and a green viability fluorescent dye (SYTO 9 dye, Thermo Fisher Scientific) [z-stacked compiled from 30 individual processed planar images; ex=642;em=661nm; pixel size=0.4; zoom=x3]."

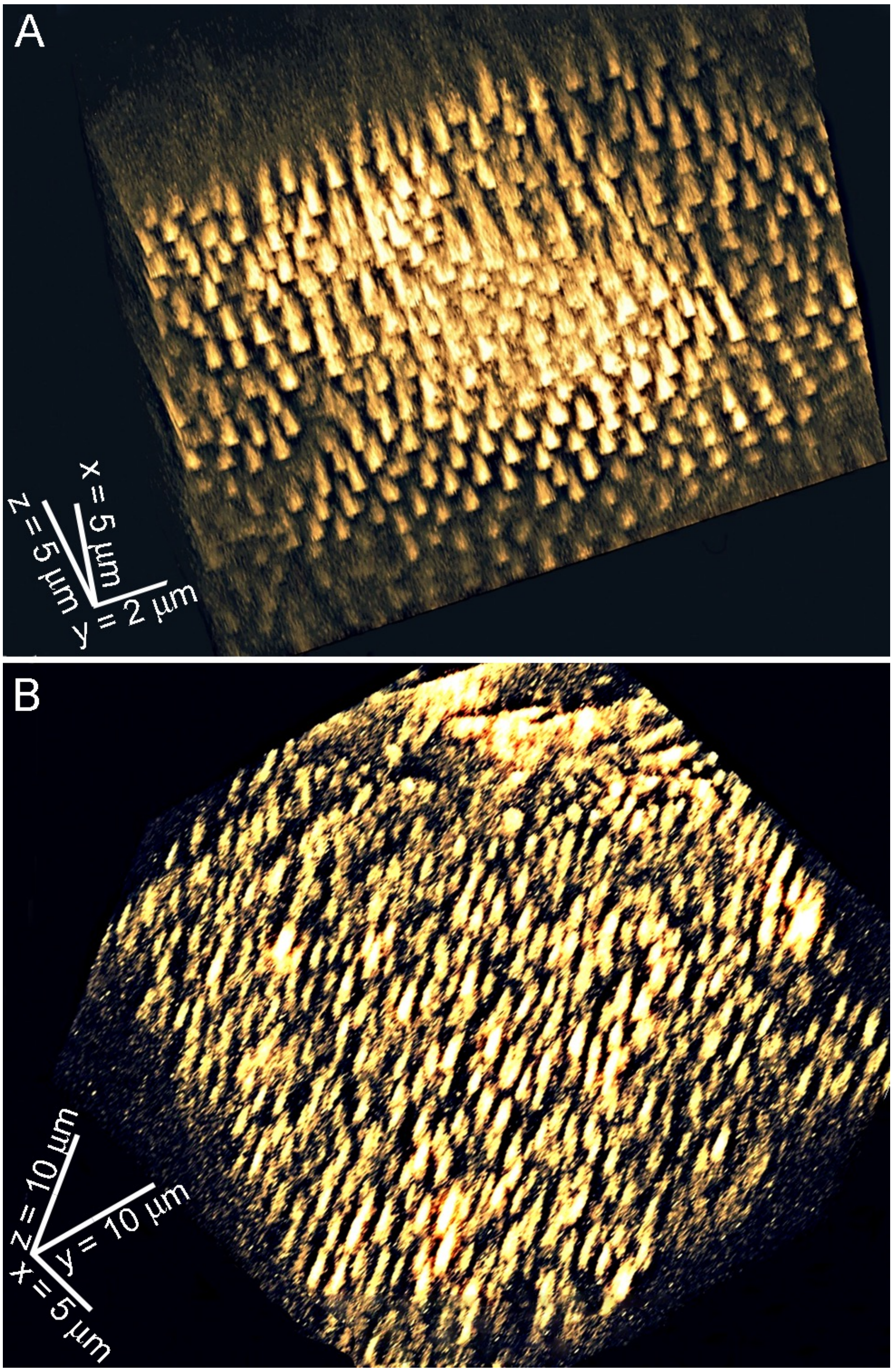

Fig. S7 Part of the future strategy for scaling-up the replication of gecko spinule surfaces in the selected material. (A,B) Laser confocal 3D digitally rendered microscope images of natural gecko skin spinule array (A) versus a polystyrene replica of the spinule array (B) to show equivalence in spinule thickness at the base and the tip and spacing in nm, as well as, shape and morphology. This technique is enabling the accurate conversion of spinule arrays into digital data formats for 3D inkjet printing. Spinules were dyed with Cell Tracker (CT) Deep Red (Thermo-Fisher Scientific: ex

= 630; em = 650) for 30 minutes in PBS, mounted onto a microscope slide then and scanned 50 times at the highest magnification, without pixellated distortion, from the top of the spinule to the base (×1000 with ×1 digital zoom: pixel size = 0.38 μm; Zeiss Confocal Laser Microscope) to generate 3D composite images [A,B = Picasa Image settings used to better resolve the spinules into clear view: sepia, cross-process, HDRish, Picasa setting; Image dimensions 22 × 22 μm; B= 22 × 22 μm].

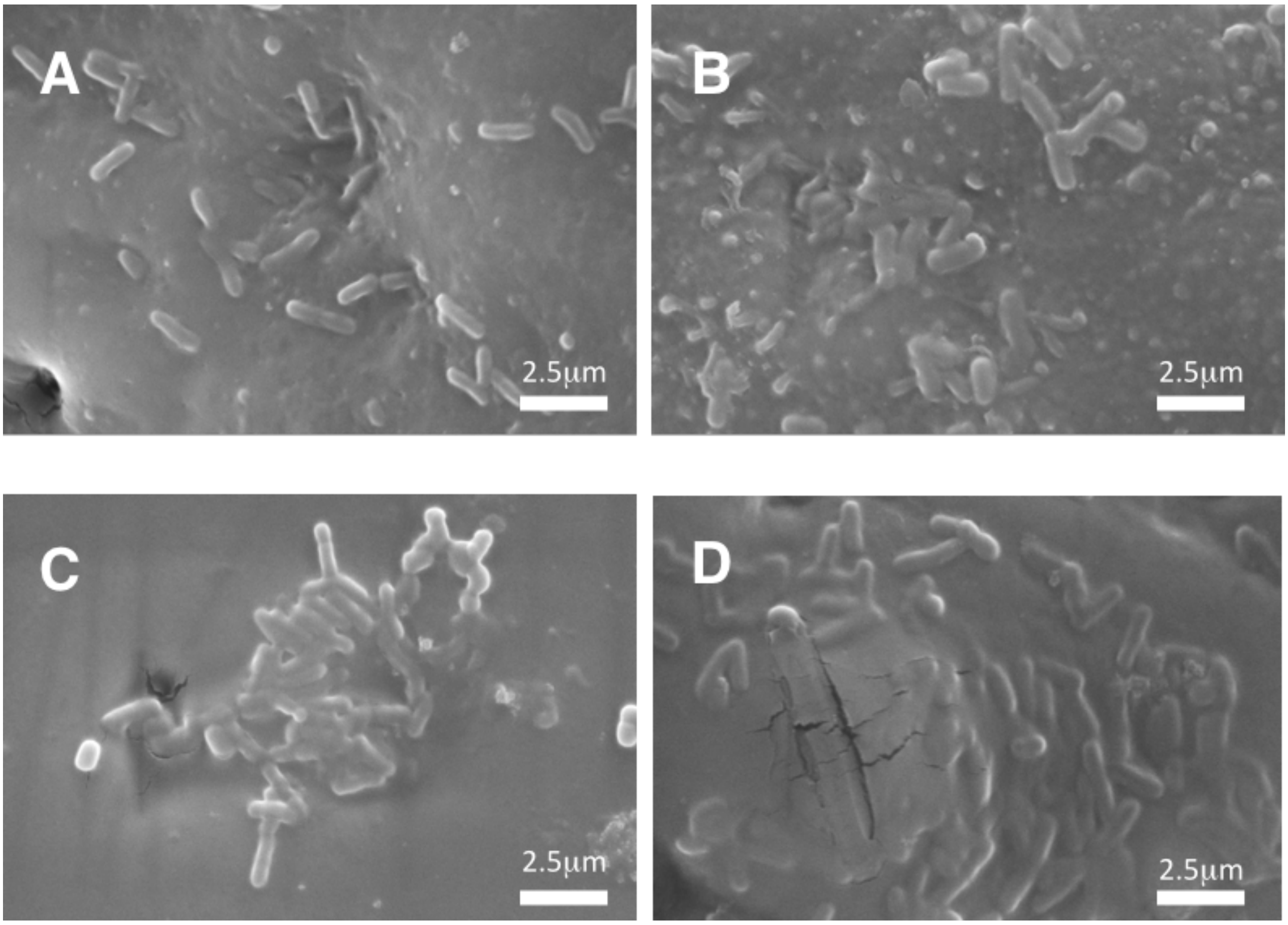

Fig. S8 SEM images of *Lactobacillus* bacteria on smooth biopolymer surfaces after 7 days of cultivation. In contrast to bacteria on biopolymeric spinules, bacteria on the smooth surfaces are fully intact, forming closely packed colonies; (A) Chitosan; (B) Alginate/ (spinules) Chitosan; (C) Chitosan/ Keratin blend; (D) Silk fibroin.

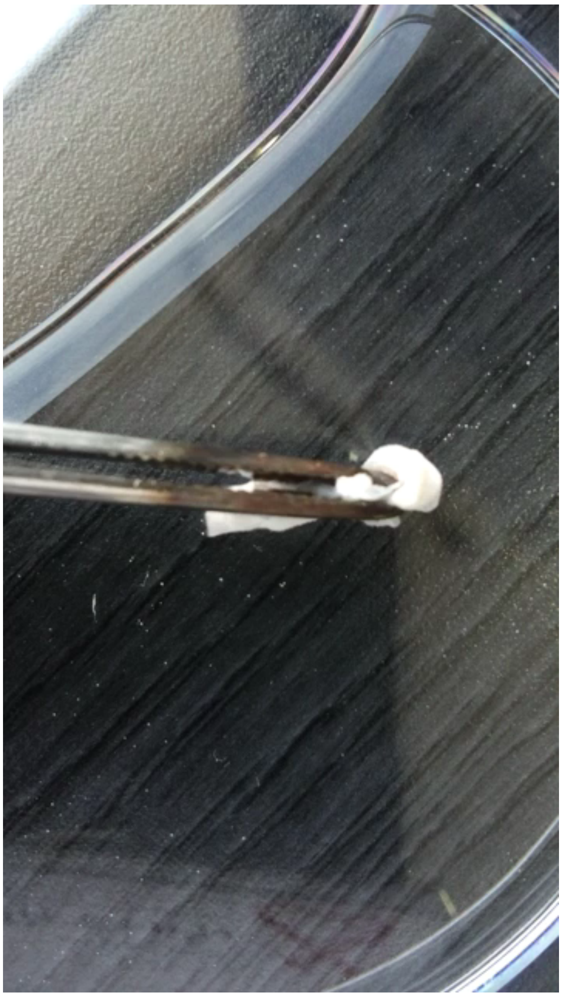

Supplementary Video 1: A short video showing the highly elastic, springy behaviour of gecko Lizard skin (shed) as it unravels and spreads itself on top of a thin-film of water. This inherent property allows shed skin to be spread evenly on to a microscope slide. After drying it remains flat with only minor undulations. The flattened panel of skin is easily molded with the impression material in stage 1 (**Fig. 1**) of the method.
